# Analysis of factors related to shoulder instability in young handball players

**DOI:** 10.1080/07853890.2021.1896439

**Published:** 2021-09-28

**Authors:** Cláudia Torres, Edgar Leitão, Joana Leal, Sara Camelo, João Paulo Sousa, Ângela Maria Pereira

**Affiliations:** a Department of Physiotherapy, Escola Superior de Saúde Egas Moniz (ESSEM), Egas Moniz Cooperativa de Ensino Superior, Caparica, Portugal; b Departamento de Desporto e Saúde, Escola de Ciência e Tecnologia da Universidade de Évora, Monte da Caparica, Portugal; c Comprehensive Health Research Center (CHRC), Monte da Caparica, Portugal; d Centro de Investigação Interdisciplinar Egas Moniz (CiiEM), Egas Moniz Cooperativa de Ensino Superior, Caparica, Portugal; e Hospital Garcia da Orta, Almada, Portugal

## Abstract

**Introduction:**

The shoulder of handball players suffers from the ongoing repetition of movement that may lead to the development of joint instability and modification of proprioceptive condition [1]. Because the articular components of the shoulder joint are considered to have extreme importance on the static and dynamic stabilisation and quality of proprioceptive information, they may compromise the athlete’s performance [2]. The purpose of our study was to verify if shoulder strength parameters (SSP) and joint position sense (JPS) of shoulder internal and external rotation may contribute to the development of shoulder instability in handball players.

**Materials and methods:**

A cross-sectional design was implemented. The sample was composed by eleven handball players of both genders (4 females and 7 males), under 18 years of age, that practiced the sport for at least 3 years. For the data collection, an isokinetic dynamometer (Biodex System 3) was used. The evaluation of SSP was implemented prior to JPS at a speed of 60°|sec (3 reps) and 180°|sec (20 reps) of internal rotation (IR) and external rotation (ER), with the shoulder positioned at 90° of abduction. The JPS was evaluated using active and passive positioning, 6 repetitions each, at 3 given external shoulder rotation amplitudes (20, 35 and 75 degrees). Prior to the data collection, all subjects signed an informed consent. This study follows all the principles of the Declaration of Helsinki.

**Results:**

For the dominant shoulder, the peak torque (PT) at 60°|sec was 41.4 Nm (±12.2) of ER and 40,6 Nm (±17.5) in IR. For the non-dominant shoulder at 60°|sec, the PT was 34.3 Nm (±10.7) of ER and 42.0 Nm (±2.8) in IR. For the dominant shoulder, the PT at 180°|sec was 37.2 Nm (±12.2) of ER and 37.9 Nm (±15.0) in IR. At the non-dominant shoulder, the PT at 180°|secr was 29.8 Nm (±10.8) of ER and 37.2 Nm (±11.6) in IR. At 60°|sec, the range of motion (ROM) was of 89.6° (±3.2) and at 180°|sec, 88.8° (±0.6). On the dominant shoulder, for the passive positioning, a difference was obtained of 6.7° (±5.8) at 75°, of 1.7° (±2.4) at 35°, and of 0° (±2.3°) at 20°. In the active positioning, a difference was obtained of 1.3° (±3.1) at 75°, of 1.0° (±3.6) at 35°, and 1.2° (±2.7) at 20°. On the non-dominant shoulder, we observed the following results, in the passive positioning, 4.7° (±3.7) at 75°, 1.3° (± 4.1) at 35°, and 0.7° (±3.5) at 20°. Finally, in the active positioning we obtained the following results: 0.5 ± 3.4 (75°), 0.7 ± 2.0 (35°) and 2.7 ± 2.4 (20°).

**Discussion and conclusions:**

Our preliminary results seem to point out that the dominant shoulder improved JPS when compared to the non-dominant. Additionally, we found that during the active positioning athletes had a greater perception of JPS, in comparison to the passive positioning. These results should help on the development of training protocols in handball, to improve shoulder proprioception and help to reduce the risk of injury. Although the results on SSP were not statistically treated, throughout the study protocol the PT seems to decrease significantly. Further studies should help to confirm our results.

## References

[1] Langevoort G, Myklebust G, Dvorak J, et al. Handball injuries during major international tournaments. Scand J Med Sci Sports. 2007;17(4):400–407. 17038157 10.1111/j.1600-0838.2006.00587.x

[2] Van den Tillaar R, Ettema G. A three-dimensional analysis of overarm throwing in experienced handball players. J Appl Biomech. 2007;23(1):12–19. 17585175 10.1123/jab.23.1.12

